# Jiří Procháska (1749–1820)

**DOI:** 10.1007/s00415-019-09371-4

**Published:** 2019-05-17

**Authors:** Michał K. Owecki, Pilar León-Sanz

**Affiliations:** 1 Department of History of Medical Sciences, Poznań University of Medical Sciences, Poznan, Poland; 2 Department of Biomedical Humanities, School of Medicine, University of Navarra, Pamplona, Spain

**Fig. 1:**
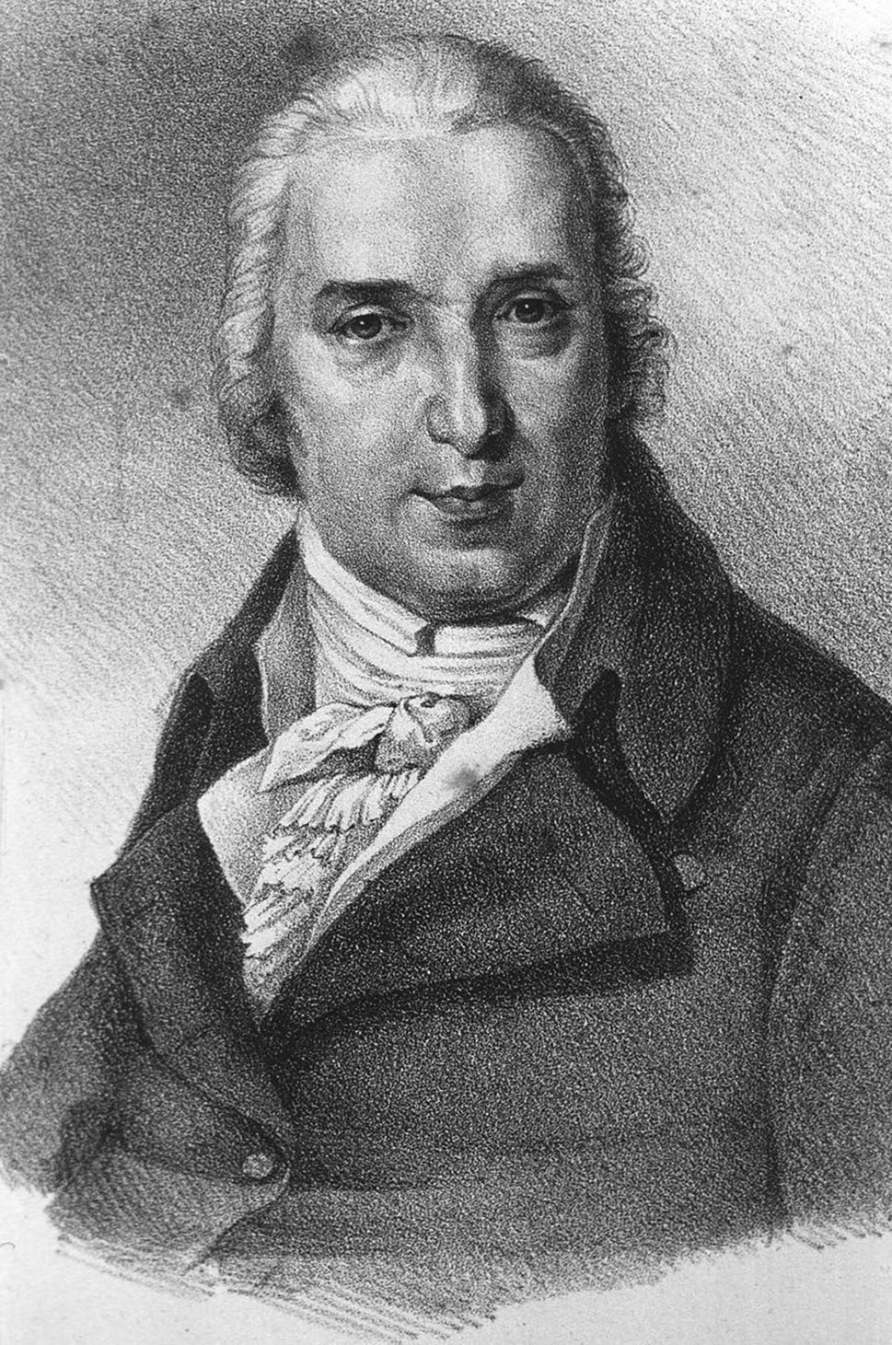
Jiří Procháska (1749–1820) [public domain]

Jiří (Georg) Procháska (Fig. 1) was born on April 10, 1749 in Blížkovice in Moravia, then a part of the Austro-Hungarian Empire, to a blacksmith's family. At the age of 6, he started his education at a local school and, as he was a good student, his parents hoped that he would choose the priesthood when he came of age. Thus, they decided to continue their son's education, first at a Jesuit junior high school in the nearby town of Znojmo, and then, from 1765, at the Faculty of Philosophy at the University of Olomouc. After graduating from the university in 1769, Procháska moved to Vienna, the capital of the Empire and the center of the country's scientific and political life. There, as he was especially interested in physiology and anatomy, he decided to study medicine [1].

Sometimes, misfortunes paradoxically have a positive effect—this was also the case in Procháska’s life: touched by disease during typhus epidemics in 1771–1772, he was hospitalized in the Clinic run by Professor Haen, a famous Viennese physician, who noticed great potential in young Procháska and his desire for knowledge. Their meeting resulted in a surprising outcome—after his recuperation, Procháska was given a job at the hospital, which provided him with sufficient financial resources to live and to study medicine. In 1774, he gained a position as a second assistant and, soon after, became first assistant to his mentor [2, 3].

In 1776, along with Haen’s death, Procháska lost his position but he continued his work with another prominent specialist of the time—professor of anatomy and ophthalmology, Josef Barth. He graduated under Barth’s supervision and, on November 9, 1776, defended his doctoral dissertation, written in Latin, devoted to issues related to urine: *Dissertatio Inauguralis Medica de Urinis*. The subject of the doctorate corresponded to the interests of the era, still heavily influenced by the dogmas of Hippocrates’ and Galen’s theory of the four humors [2–4].

Procháska's research work resulted in a number of publications shortly after graduation. In 1778, he published a treatise on the anatomy and physiology of the circulatory system. In the same year, he published his first dissertation addressing neurological issues: *De Carne Musculari Tractatus Anatomico-Physiologicus*. In this dissertation (in Latin), he tried, in an interesting way, to explain the physiology of skeletal muscle contraction. Procháska hypothesized that contraction of a muscle is a consequence of the expansion of blood vessels, enabling an abundant supply of blood to the muscle tissue. According to his concept, increased blood flow to the muscle led to distortion of the muscle fibers, eventually provoking their contraction, and thus the contraction of the entire muscle [5]. His conception reflected an evolution toward an iatromechanical approach, which explained the movement of the human body, and that of all living creatures, according to the rational mechanics of the eighteenth century.

On March 29, 1778, Procháska was promoted to *magister* of ophthalmology. In the same year, Procháska was offered a job at the University of Prague, where he attained the position of professor of anatomy and ophthalmology [2]. While he was in Prague, in addition to other works, Procháska produced his most important neurological publications. In 1779, a Latin dissertation on the structure and function of the nerves appeared [6]. A few years later, in 1784, he published his monumental work on the physiology of the nervous system, partly based on his previous treatise [7]. In continuity with the iatromechanical approach to this problem, Procháska introduced a new concept of “nervous power” (“vis nervosa”) in this work. “Nervous power", hidden as potential energy in the nerves, provokes a response when reaching the muscle—contraction. The area of the nervous system that initiated motor stimulation in response to sensory arousal was supposed to be the “sensorium commune”, which, according to his conception, included the spinal cord, medulla, the cerebral and cerebellar peduncles, and part of the thalamus. For iatrophysicists, the forces that acted in a living organism were external—the sensation was explained by means of a fibrous model: the nerves were very sensitive, highly irritable fibers that communicated the jolts caused by the turbulence of a movement to the brain [8]. Moreover, Procháska assumed the existence of sensory nerve fibers (afferent) and motor fibers (efferent), almost 3 decades prior to the famous discovery by the Scottish neurophysiologist Charles Bell (1774–1842) [9]. Procháska also discovered through experiment and reported in his book that the brain and consciousness were not needed for the reflex response; only the spinal cord.

After Professor Barth’s resignation in 1791, Procháska returned to Vienna, taking up a position after his former mentor and promoter. As professor of anatomy, physiology and ophthalmology, he worked until his retirement in 1819. Procháska continued his research in Vienna, which resulted in further works in the field of anatomy and physiology, but he did not return to strictly neurological issues. Among these publications, the most important was a textbook on physiology, translated into several languages over the course of the nineteenth century. During his time in Vienna, Procháska created an imposing collection of anatomical preparations, later bought by the government for the enormous sum of 6000 florins. From 1805, he focused primarily on physiology, entrusting anatomical issues to his prosector, Michael Mayer. In addition to his scientific work, Procháska also ran a famous ophthalmology practice—widely regarded as an outstanding Viennese specialist, he carried out over 3,000 cataract surgeries. At the same time, he was a man of great heart—he treated and operated the poor for free [1–3].

As well as his medical and university work, Procháska also participated in the activities of several scientific societies—in Prague, Nancy and St. Petersburg. He was not only a good teacher, but also a great organizer—for example, on the occasion of the visit of the Emperor Joseph II to Prague in 1783, Procháska gained funds to renovate the seat of the anatomy faculty at the local university [1–3].

Not much is known about Procháska's private life. In 1779, he married Anna Štěpánská, daughter of a Prague councilor, with whom he had a daughter, Emilia, in 1792. His wife passed away in 1810, at the age of 49. Procháska probably also had some artistic interests—for example, it is recorded that, while on honeymoon, he was involved in painting and music. He died in Vienna, at about 7 O’clock in the morning, on July 17, 1820, and was buried in St Mark’s Cemetery [3, 10].

Jiří Procháska was an ophthalmologist, physiologist, and anatomist but it is impossible to consider him a neurologist. Paradoxically, however, his contribution to the development of the emerging field of neurology is undeniable. His theory of the reflex, with its afferent and efferent branches and a decision-making center in between, became one of the foundations of clinical, experimental, and theoretical neurology in the century to follow.
